# Longitudinal virological outcomes and factors associated with virological failure in behaviorally HIV-infected young adults on combination antiretroviral treatment in the Netherlands, 2000 to 2015

**DOI:** 10.1097/MD.0000000000016357

**Published:** 2019-08-09

**Authors:** Annouschka M. Weijsenfeld, Charlotte Blokhuis, Martijn M. Stuiver, Ferdinand W.N.M. Wit, Dasja Pajkrt

**Affiliations:** a Department of Paediatric Infectious Diseases, Emma Children's Hospital, Amsterdam UMC, University of Amsterdam; b Department of Internal Medicine, Amsterdam UMC, University of Amsterdam; c Department of Clinical Epidemiology Biostatistics and Bioinformatics, Amsterdam UMC, University of Amsterdam; d HIV Monitoring Foundation (Stichting Hiv Monitoring), Amsterdam, the Netherlands.

**Keywords:** combination antiretroviral therapy, HIV, virological failure, young adults

## Abstract

Achieving and maintaining viral suppression in young adults (18–24 years) living with HIV is challenging. Overall HIV viral suppression rates are lower in young as compared to older adults. Longitudinal data provide valuable insight on dynamics of viral suppression and variables of potential influence on HIV virological failure (VF), but is scarce in young adults living with HIV on combination antiretroviral therapy **(**cART). We evaluated longitudinal virological outcomes of behaviorally young adults (18–24 years) living with HIV in the Netherlands over a period of 15 years.

We analyzed data from the Dutch national HIV database of 816 young adults living with HIV on cART in the Netherlands from 2000 to 2015. VF was defined as 2 consecutive detectable plasma HIV-1 viral load (VL) measurements > 200 copies/ml. Generalized linear mixed model analyses were used to assess HIV VF over time and identify risk factors associated with VF.

VF during the study follow-up occurred at least once in 26% of cases. The probability of experiencing VF decreased over the study period per calendar year (OR 0.78, 95% confidence interval [CI];0.72; 0.85). Factors significantly associated with VF were being infected through heterosexual contact (OR 5.20, CI 1.39;19.38) and originating from Latin America or the Caribbean (OR 6.59, CI 2.08;20.92). Smaller, yet significant risk factors for VF were being infected through a blood transfusion or a needle accident (OR9.93, CI 1.34;73.84, and having started with cART with a nadir CD4 count >500 cells/μl (OR 11.36, CI 2.03;63.48).

In our large cohort of young adults, the risk of VF has diminished over 15 years. Specific subgroups were identified to be at risk for suboptimal treatment.

## 1 Introduction

Since the introduction of combination antiretroviral therapy (cART), the life expectancy and future perspectives of people living with HIV have improved impressively. Availability of cART and improved treatment options have revolutionized HIV infection into a chronic condition, with optimal HIV viral suppression and immunological recovery as most important determinants for long term optimal health outcomes.^[1]^ Additionally, HIV viral suppression lowers the chance of transmission to minimum levels, turning HIV treatment into an important preventive measure.^[2]^

The subgroup of young adults within the HIV population show lower rates of achieving and maintaining HIV viral suppression as compared to older patients.^[3]^ As such, they are at increased risk for the development of drug resistant mutations reducing future treatment options.^[4]^ The development of independence, self-consciousness, and autonomy (hallmarks of adolescence and young adulthood) threaten optimal and durable treatment of all young adults that have to cope with a chronic condition.^[5,6]^ Missed clinic appointments, lower rates of retention in care, and stigma-related problems like secrecy, occur frequently and hamper optimal treatment adherence in young adults living with HIV.^[7,8]^

As of 2016, approximately 30% of new HIV infections worldwide occurred in adolescents and young adults 15 to 25 years of age.^[9]^ In the Netherlands, 7% to 14% of new HIV diagnoses in 2016 were in young adults.^[10]^ Acknowledging the need for research that provides insight into the effect of HIV treatment in this specific age group, several studies have been conducted. Cross-sectional studies from the United States report relatively low HIV suppression rates from 63.6% to 74% in young adults living with HIV on cART, as compared to 81% in older adults.^[3,11]^ Maintenance of viral suppression is even more challenging in young adults as well as in adolescents, as reflected by higher rates of virological rebound and lower durable viral suppression rates as compared to older adults.^[3,12]^ Factors associated with effective viral suppression were consistent linkage to HIV care, >6 months of antiretroviral treatment (ART) and >90% adherence.^[11]^

A 2014 systematic review and meta-analysis on medication adherence levels in 53 countries worldwide among adolescents and young adults living with HIV, described adherence levels of 62% in Europe. The authors noted the paucity of longitudinal studies, as they could only assess cross-sectional data.^[13]^ Longitudinal results derived from a small study in adolescents and young adults (aged 15–22 years) showed that the medium time from cART initiation to non-adherence was 12 months and maintenance of an undetectable viral load (VL) was seen in only 50% of patients.^[14]^

Although cross-sectional studies provide important information on viral suppression as a reliable indicator of adherence, this approach does not provide information on dynamics of virological failure (VF) and risk factors associated with VF over time. Up-to-date research on viral suppression in young adults living with HIV is concentrated in the United States, and scarce in Europe or the Netherlands. As the organization and management of HIV care in young adults may differ between continents, European longitudinal results are needed to improve young adult related care. A review article of the International Aids Society highlighted the importance of using the WHO definition of adolescents and young adults in research (10–24 years of age), and differing between adolescents (10–18) and young adults (19–24) as variations in age ranges defining adolescence and young adulthood complicate comparisons and generalization of study findings.^[15]^

Therefore, we evaluated longitudinal virological outcomes of behaviorally young adults (18–24 years) living with HIV in the Netherlands over a period of 15 years. Secondly, we explored factors associated with VF to identify at-risk subgroups within this population.

## 2 Methods

We performed a multicentre retrospective cohort study using data from the ATHENA cohort. The ATHENA cohort is managed by Stichting HIV Monitoring (SHM) (January 1998–December 2015). A detailed description of the ATHENA cohort objectives, methodology, and procedures has previously been described.^[16]^ At the start of data collection all patients who are registered in the Stichting HIV Monitoring (SHM) database gave permission for the use of their data for medical research. Patients were eligible for inclusion in the study if they were

1. diagnosed with an HIV-1 infection,
2. linked to HIV care in one of the 26 adult HIV treatment centers in the Netherlands for at least 2 consecutive visits between the age of 18 and 25 years, and
3. were prescribed cART for at least 6 months.

In accordance with WHO guidelines, we differed between adolescence and young adulthood and chose 18 to 24 years as the age range for young adults, consistent with the age of 18 marking legal adulthood and transfer to adult care in the Netherlands. The observation period thus started for

1. persons already in clinical care before their age of 18 years, at the age of 18 years;
2. patients newly entering into care between the age 18 and 24 years, at the first available health care visit between the age of 18 and 24 years.

We chose to perform statistical analyses on data from the late cART era, collected from the year 2000 on, to minimize the effect of availability of different (or less potent) cART regimes over time. The following data were collected: age, gender, country or region of origin, HIV transmission route, age at HIV diagnosis, years on anti-retroviral therapy, plasma HIV VL measurements, peak VL, and nadir CD4+ count. VL measurements were routinely collected during outpatient clinical visits every 3 to 6 months, and more often in case of a detectable VL. Virological data were collected until the age of 25 years or the study closure date (December 2015), whichever came first. We defined cART as either a minimum of three antiretroviral drugs from at least 2 classes,^[17]^ or the use of three nucleoside reverse transcriptase inhibitors (NRTIs) as a proxy for cART.^[18]^ If the first start date of cART was unknown (as was sometimes the case in migrants already using cART when entering into care in the Netherlands), we started data collection from the first exact known date of cART prescription to guarantee an adequately documented minimum of 6 months of cART. We excluded patients who were perinatally infected, and although a small proportion (3.9%) of the patients was infected through a blood transfusion or a needle accident, we used the term behaviorally infected for every non-perinatally infected patient. Further, post-pregnancy observations of women with high nadir CD4+ T-cell counts (>350 cells/μl) were excluded, as VF in this group is biased by planned discontinuation of cART according to them current guidelines for pregnant and post-pregnant women during a large part of the study period.^[19]^

## 3 Statistical analyses

Descriptive statistics were used for baseline demographic characteristic and laboratory variables. All characteristics were expressed as medians (IQR) or frequencies and percentages, where appropriate. VL measurements were based on assays between 2000 and 2015 using different lower limits of detection (ranging from < 500 copies/ml in the year 2000 to < 20/40 copies/ml in 2015). VF was defined as 2 consecutive detectable HIV VL measurements > 200 copies/ml or above the lower limit of detection where appropriate. HIV VL measurements were considered undetectable in case of HIV VL levels lower than the lower limits of detection at the time of the measurement. Missing values on nadir CD4 count (8.7%) and peak logVL measurements (10.8%) were imputed by automatic multiple imputation techniques using linear regression and fully conditional specification. The imputation model was constructed following the recommendations by van Buuren, including all variables in the imputation model.^[20]^ Imputed values were checked for credibility. Results of five imputed datasets were pooled by combining the estimates and variance from all five imputed datasets according to Rubin rules.^[21]^

To assess aforementioned socio-demographic and HIV-related variables as potential risk factors for VF, we used a multivariable generalized linear mixed (logit) model which handles missing values, loss to follow up and repeated measurements nested within a single subject. The model was fit by maximum likelihood. Subjects were included as random effects, allowing a random intercept per patient to account for repeated observations. All other variables were treated as fixed effects. The variables assessed for potential influence on VF were calendar year (as a categorical variable), gender, region of birth, HIV transmission route, nadir CD4+ count, peak VL and cumulative duration of antiretroviral therapy use. A sensitivity analysis was performed to assess the impact of triple NRTI use on the results.^[22]^ Results are reported as odds ratios (OR) with 95% confidence intervals (CI). A *P* value < .05 was considered statistically significant. Statistical analyses were performed using R (Version 1.0.143, R Foundation for Statistical Computing, Vienna, Austria 2014), and SPSS (IBM SPSS Statistics for Windows, Version 24.0. Armonk, NY: IBM Corp 2016).

## 4 Results

**Figure 1:**
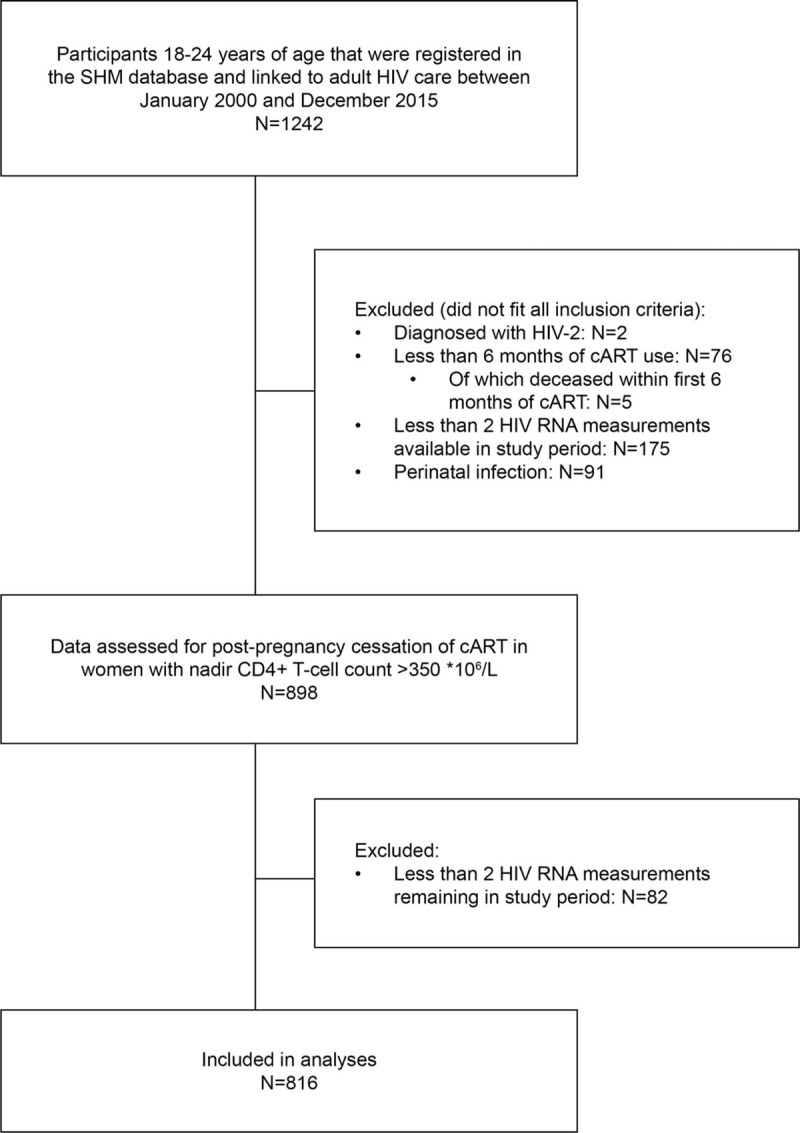
Flowchart describing the in- and exclusion of study participants.

A total of 1242 patients received care at an adult HIV treatment centre during young adulthood, and 816 were included in this study (Fig. 1). Most participants were HIV infected via heterosexual (389, 47.7%) or men-who-have-sex-with-men contact (MSM; 349, 42.8%). The majority of patients originated from the Netherlands (309, 37.9%), Sub-Saharan Africa (303, 37.1%) or Latin America and Caribbean Islands (131, 16.1%). Patients were HIV diagnosed at a median age of 20.6 years (18.9–22.0). cART was the initial therapy for the majority of patients in our cohort, and was started at a median age of 21.7 years (19.9–22.9). Of the 816 patients, 74 (9.1%) dropped out during the study period, of whom 13 (1.6%) moved abroad and 50 (6.1%) were lost to follow-up. The median follow-up time was 1.5 years (0.8–2.9). Of all included patients, 11 (1.3%) died before the age of 25 years (Table 1). Causes of death were acquired immune deficiency syndrome (AIDS) (n = 5), non-natural death (n = 3) and other causes (n = 3).

**Table 1:**
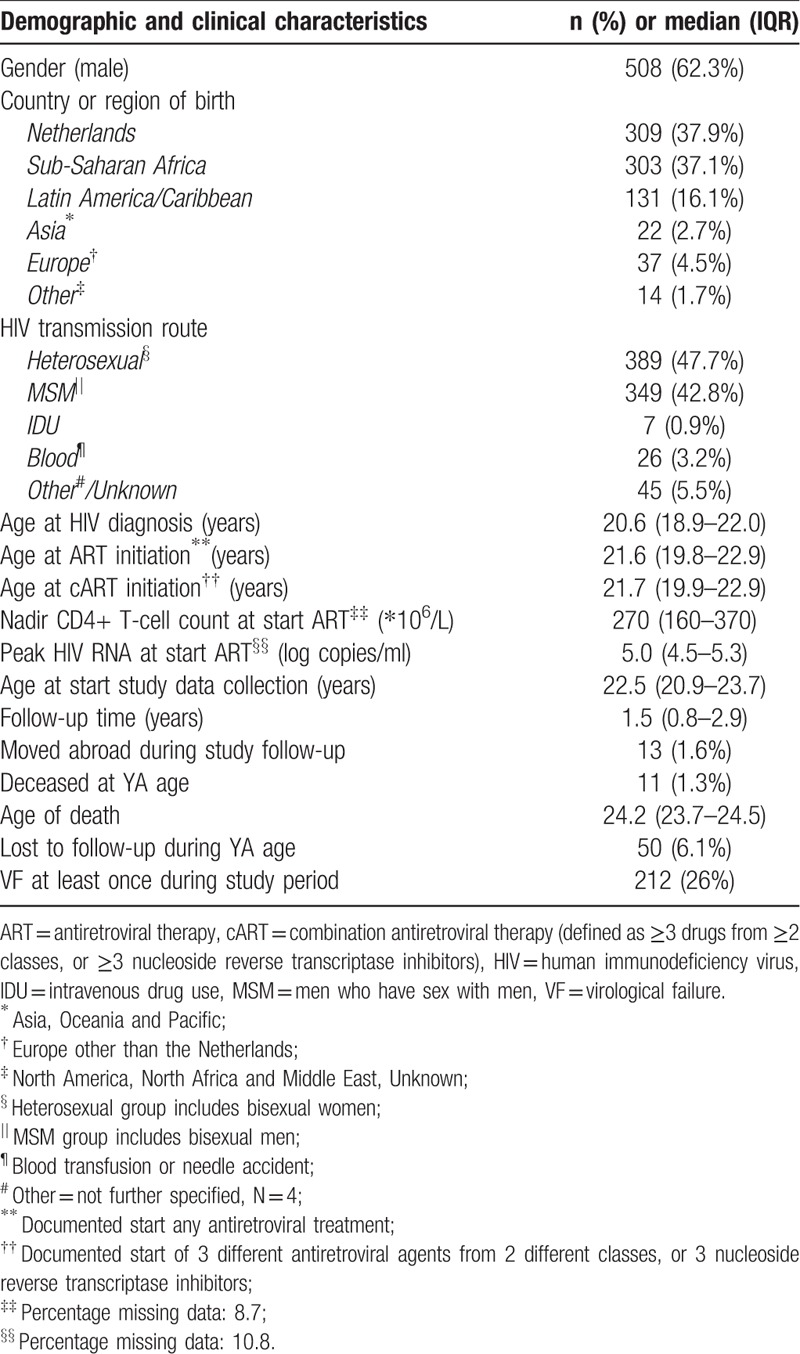
Demographic and clinical characteristics of behaviourally HIV-infected young adults (N = 816) in the Netherlands between 2000 and 2015.

VF occurred in 26% of participants at least once during the study follow-up. The probability of experiencing VF decreased per calendar year (OR 0.78, 95%CI 0.72/0.85). VF was more probable in heterosexually infected patients (OR 5.20, 95% CI 1.39;19.38), patients originating from Latin America or the Caribbean (OR 6.59, 95% CI 2.08;20.92) and patients infected through a blood transfusion or a needle accident (OR 9.93, CI 1.34;73.84. Patients with a nadir CD4+ count >500 cells/μl were more likely to experience VF (OR 11.36, 95%CI 2.03;63.48). We found no associations between VF and gender, Sub-Saharan African origin, peak HIV VL, or cumulative duration of ART use (Table 2). Separate analyses of complete cases only (without imputation N = 711) showed an increased risk for VF in patients originating from Sub-Saharan Africa (OR 3.80, CI 1.08;13.41, *P* = .04), which was not observed in the imputed datasets. Contrary, no additional risk was observed in complete cases for patients infected through a blood transfusion or a needle accident (OR 7.95, CI 0.64; 98.05, *P* = .11). All other outcomes were similar (data not shown).

**Table 2:**
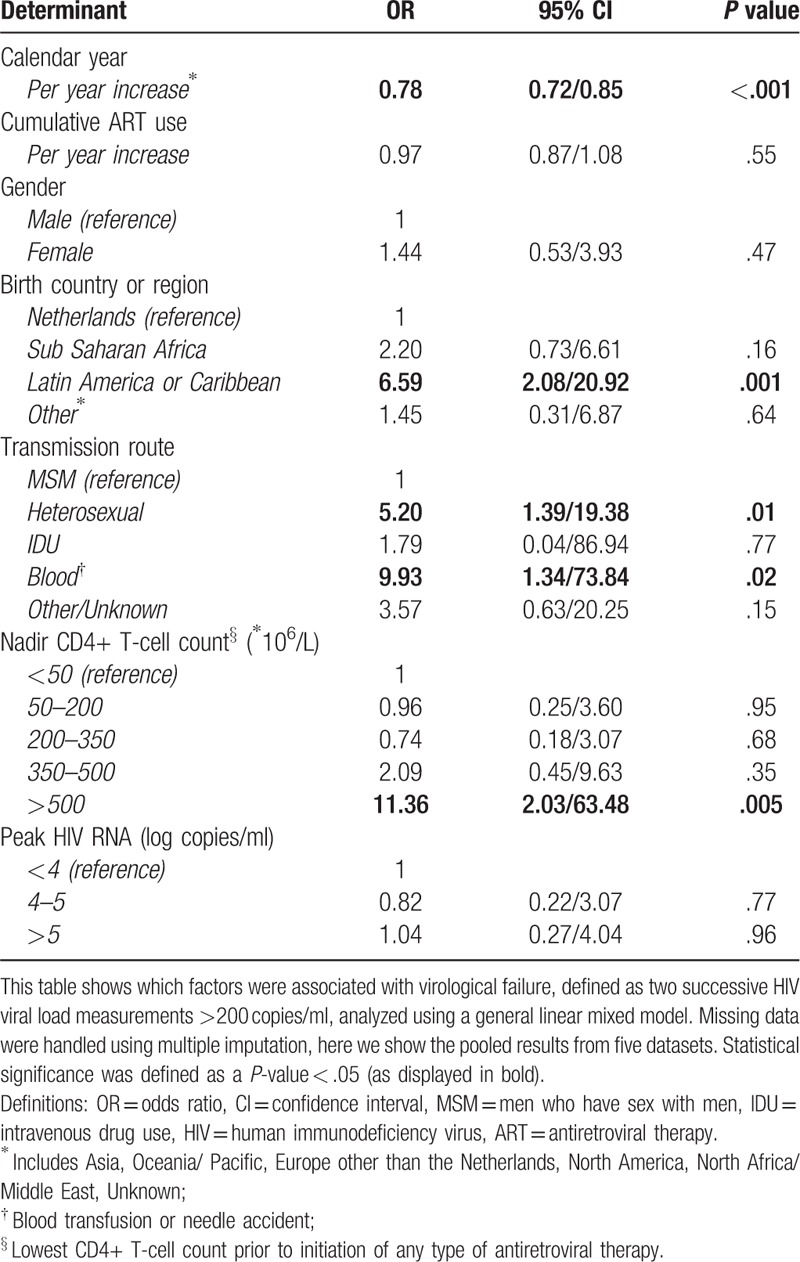
Associations with virological failure.

**Figure 2:**
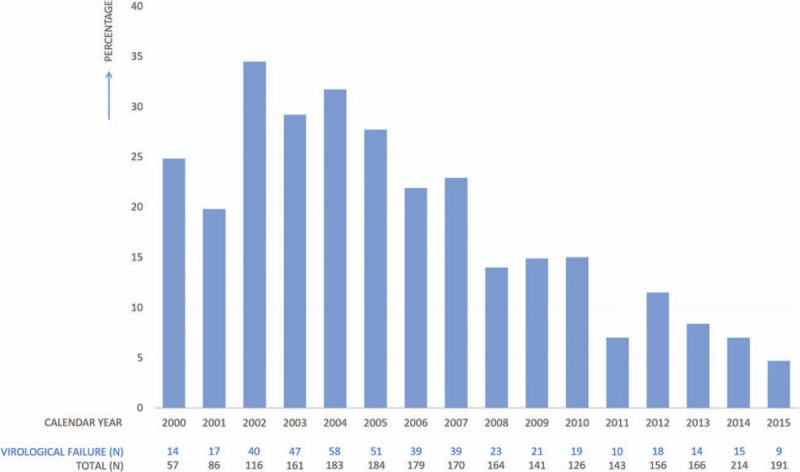
Proportion of young adults (18–24 years) experiencing virological failure per calendar year. This graph represents a cross-sectional view of the percentage of study participants experiencing virological failure per calendar year. The absolute numbers of participants for which data was available per year are displayed below the graph.

The percentages of cases experiencing VF sorted by calendar year are shown in Figure 2. Repeating the analysis taking into account the use of a triple NRTI regimen had no significant impact on the results (data not shown).

## 5 Discussion

Our study assessed long-term virological outcomes in behaviorally young adults living with HIV on cART in the Netherlands. In our cohort, 26% of 816 participants were not sustained virological suppressed during the study follow-up after starting cART. Outcomes improved significantly over time, highlighting the importance of taking into account calendar year when assessing and interpreting HIV-related outcomes for young adults. During our study period, some important milestones in cART development occurred, in 2006 the first 1 pill regime was introduced, followed by the introduction of HIV integrase inhibitors in 2007 and the second-generation HIV integrase inhibitors in 2013. Our findings suggest that young adults greatly benefitted from improved cART regimens, that have shown to be more potent and forgiving with lesser side effects.^[23]^

We report higher overall HIV suppression rates than results from cross-sectional studies in young adult populations in the United States (53–64%) and in adolescents and young adults in Europe (62%).^[11,13]^ This could, in part, be explained by methodological factors, including the use of different measures of adherence, such as viral suppression, pill count, and self-report.^[13]^ However, we believe that longitudinal data provide a more thorough view on treatment outcomes by using multiple observations over time.

A 4-year study on trends of antiretroviral therapy prescription and viral suppression in young adults reporting HIV non-suppression rates of 20% to 30% approached our results more closely. In accordance with our results, this study reported on the increased proportion of sustained viral suppression in young adults over the years.^[24]^ A recent longitudinal study with a median follow-up period of 2.1 years from the United States described VF in 29% in those previously achieved viral suppression. In line with our data, young adults in this study who entered care after the year 2012 showed significantly better outcomes with only 3% experiencing VF after start cART.^[25]^

Despite these similarities, our results indicate that young adults living with HIV in the Netherlands have generally better virological outcomes than their peers in other high-resource countries. This may be caused, in part, by differences in general access to health care and/or organization of HIV care in Western countries. Today, HIV suppression rates in young adults in our cohort are comparable to reported suppression rates of 95% in adults in the Netherlands in 2016.^[10]^ Previous studies in young adult and older adult populations living with HIV report older age as a contributing factor to treatment adherence.^[26,27]^ In our cohort, development of young adults towards adulthood might be of influence on improved outcomes as well. However, taking into account the large effect of calendar year, we have to consider the possibility that in today's cART era, adherence rates in young adults further improved due to more potency, less side effects, and higher availability of current 1-pill regimens as compared to older treatments. Furthermore, with newer regimens, the necessary rate of adherence to achieve viral suppression may be lower than with older regimens.^[28]^

We identified several subgroups and factors that are associated with VF in our cohort. Firstly, we found a strong association between VF and being heterosexually infected as compared to being infected though MSM contact. Poorer treatment outcomes in heterosexuals as compared to MSM were previously observed in studies in both adults and young adults.^[29,30]^ Studies suggest that acceptance of same sex relationships within a society is likely of considerable influence on access to HIV care. Moreover, young MSM with less negative feelings towards homo- and bisexuality and HIV infection are more likely to be engaged in care.^[31,32]^ In 2015, the Dutch Association of People living with HIV published a study on quality of life in 468 people living with HIV in the Netherlands, of whom 40% was heterosexual. Heterosexuals reported more HIV-related depressive symptoms and feelings of guilt and shame as compared to the MSM group. Further, heterosexual men were less likely to seek support from family or friends in coping with their HIV infection.^[33]^ Our results may thus (at least partly) be explained by these psychosocial components that have a negative effect on adherence.^[34]^ VF occurred more often in young adults infected though a blood transfusion or needle accident. This association reflects a small subgroup and we have to consider unmeasured factors influencing optimal HIV suppression in this group. As HIV infection through a blood transfusion in the Netherlands occurred mainly in the beginning of the epidemic, this group was most likely exposed to different (less potent) cART regimes and has a high chance of having developed multiple drug resistance. Further, for persons not originating from the Netherlands, HIV transmission routes cannot always be verified.

In several Western cohorts, treatment outcomes were worse for people with a migrant background as compared to their native counterparts.^[35,36]^ These differences occur also within the MSM population.^[29,37]^ We could not confirm associations between VF and originating from Sub-Saharan Africa (SSA)^[36]^ with high confidence and observed differences between complete case and imputed case analyses. The weak effect that was found in the complete case analyses for people originating from SSA was attenuated in the imputed cases datasets losing statistical significance. Most likely, a substantial proportion of cases originating from SSA already started on (c)ART before immigration to the Netherlands. Imputing missing baseline values within the group originating from SSA might therefore have led to loss of significant association between VF and originating from SSA. Hence, longer survival on therapy can explain their generally better virological outcomes.

We did find that young adults originating from Latin America or the Caribbean were at higher risk for VF. People living with HIV in the Netherlands originating from non-Western regions were less likely to have disclosed their status and more likely to experience self-stigma as compared to people living with HIV originating from the Netherlands or other Western countries. Additionally, financial problems are most often reported in this subgroup.^[33]^ These psychosocial determinants as well as the presence of socio-economic inequality may contribute to the increased risk for VL in young adults originating from Latin America or the Caribbean. Details on socio-economic disadvantages, such as financial problems, unemployment, unstable housing, and low level of education, were not available to us, but were found previously to be strongly associated with adherence and viral suppression.^[38,39]^

Our study focused on clinical and socio-demographic characteristics, and future research is needed to gain better insight into the representation of identified risk factors for VF. Factors as self-efficacy, self-motivation, outcome expectation, social support, substance use, mental health problems, and perceived stigma may contribute to treatment adherence differently within specific YA subgroups at risk for VF.^[14,34,40]^

Starting cART at high nadir CD4 levels was identified as a risk factor for VF. Although this phenomenon is hard to explain, there is a possibility that it is driven by the lack of feeling for urgency and necessity for cART at this asymptomatic stage.^[34]^ Further analyses are needed to assess whether this effect holds in current times, in which it is more common to start treatment regardless of CD4 count.^[41]^

Although we assessed long-term outcomes with robust statistical analyses of a large cohort of young adults in the Netherlands, our study has some limitations. Our study covers a long time- period in which the lower limit of detection of HIV VL in a sample of blood decreased. Therefore, determination of VF around the year 2000 was less accurate than in more recent years. As a result, the actual effect of age and calendar year is probably stronger, as the incidence of VF might be underestimated during the first time- period of the study.

Many potentially influencing social and personal factors were not available. Additionally, we included only those patients who were registered in the SHM database and prescribed cART (respectively 97.9 and 91.8% of all people living with HIV in the Netherlands^[10]^). Since linkage to care and treatment use are relevant predictors of virological suppression,^[42]^ the actual number of young adults living with HIV that do not achieve viral suppression is probably higher than reported in our study.

In summary, in this longitudinal cohort study, we demonstrated that VF occurred in 26% of young adults living with HIV in the Netherlands. Heterosexual infection, Latin American or Caribbean origin, and nadir CD4 count >500 cells/μl at therapy initiation were additional factors that increased the risk of VF in young adults. Assessing group characteristics of those more vulnerable to suboptimal treatment to reveal what is driving adherence problems is crucial as well as identifying factors that promote treatment success.

## Author contributions

**Data curation:** Ferdinand Wit.

**Formal analysis:** Annouschka Weijsenfeld.

**Methodology:** Annouschka Weijsenfeld, Martijn Stuiver, Ferdinand Wit.

**Supervision:** Martijn Stuiver, Dasja Pajkrt.

**Validation:** Charlotte Blokhuis.

**Visualization:** Charlotte Blokhuis.

**Writing – original draft:** Annouschka Weijsenfeld.

**Writing – review & editing:** Charlotte Blokhuis, Martijn Stuiver, Ferdinand Wit, Dasja Pajkrt.
